# Establishing the UK DNA Bank for motor neuron disease (MND)

**DOI:** 10.1186/s12863-015-0236-6

**Published:** 2015-07-14

**Authors:** Lucy Smith, B. C. Cupid, B. G. M. Dickie, A. Al-Chalabi, K. E. Morrison, C. E. Shaw, P. J. Shaw

**Affiliations:** Motor Neurone Disease Association, PO Box 246, Northampton, NN1 2PR UK; NIHR Biomedical Research Unit in Dementia, Department of Clinical Neuroscience, King’s College London, London, SE5 8AF UK; Institute of Biomedical Research, College of Medical and Dental Sciences, University of Birmingham, Birmingham, B15 2TT UK; Sheffield Institute for Translational Neuroscience, University of Sheffield, 385A Glossop Road, Sheffield, S10 2HQ UK

**Keywords:** Motor Neurone Disease (MND), Amyotrophic Lateral Sclerosis (ALS), Biobank

## Abstract

In 2003 the Motor Neurone Disease (MND) Association, together with The Wellcome Trust, funded the creation of a national DNA Bank specific for MND. It was anticipated that the DNA Bank would constitute an important resource to researchers worldwide and significantly increase activity in MND genetic research. The DNA Bank houses over 3000 high quality DNA samples, all of which were donated by people living with MND, family members and non-related controls, accompanied by clinical phenotype data about the patients. Today the primary focus of the UK MND DNA Bank still remains to identify causative and disease modifying factors for this devastating disease.

**Electronic supplementary material:**

The online version of this article (doi:10.1186/s12863-015-0236-6) contains supplementary material, which is available to authorized users.

Motor Neuron Disease (MND) is a fatal, rapidly progressive disease that affects the brain and spinal cord and which ultimately leads to respiratory failure around 2–5 years following symptom onset [1, 2]. Approximately 1 in 300 people develop MND but its prevalence is low, at about 6–8 in 100,000 because of short life expectancy [3]. There is no diagnostic test and treatment is largely palliative, with only one agent, riluzole, having a modest effect in extending survival. Genetic factors undoubtedly play a role in most cases of the disease, both in pathogenesis and rate of progression, with about 5–10 % of all patients having a clear family history of MND and in some cases, frontotemporal dementia [4–6]. Over 100 genes have now been implicated in the causation of MND [7]. No consistent environmental risk factor has been identified, although it is possible that such factors may trigger disease in genetically susceptible individuals, and therefore it is plausible that apparent sporadic cases of MND will be genetically determined to some degree [8, 9].

An essential starting point for successful genetic research is access to high quality samples, accompanied by detailed clinical information. Large-scale gene sequencing and association studies need many thousands of samples to be screened such that results are statistically significant. Access to such samples had become a major obstacle in exploring the pathogenesis of MND and the concept of an MND DNA Bank was born. The objectives of the initial study were threefold: 1) To collect cohorts of patient, parent/sibling and control samples from sporadic and familial MND; 2) To collect clinical information in order to examine susceptibility traits in clinical subgroups of MND; 3) To make this resource available to the international research community and to foster collaboration between research teams, in order to identify genetic risk factors for MND.

## Organisational structure of the UK MND DNA Bank

The UK MND DNA Bank was a collaborative project adopting a ‘Hub and Spoke’ model, with three regional ‘Hub’ centres linking with a total of 16 ‘Spoke’ centres across England, Wales, Scotland and Northern Ireland (Table 1). Recruitment to the study and sample collection was coordinated at the Hub centres by a DNA Bank Co-odinator based in London. Samples were obtained from sporadic and familial MND patients attending MND clinics in the UK, their spouses (or other genetically unrelated controls) and blood relatives by hub centre-based nurses. A DNA Bank research nurse was affiliated to each Hub centre to act as patient liaison, collect clinical information from patients, controls and family members, and take blood samples. Samples within the UK MND DNA Bank are housed at CIGMR Biobank, at the University of Manchester. In addition, as one of the Public Health England collections, the European Collection of Cell Cultures (ECACC) manages the transformation and storage of EBV-transformed lymphocytes derived from blood samples from participants providing an everlasting supply of DNA for the Bank.

**Table 1:** Hub and spoke model for sample collection

King’s College Hospital, London	Queen Elizabeth Hospital, Birmingham	Royal Hallamshire Hospital, Sheffield
The National Hospital for Neurology and Neurosurgery, and Royal Free Hospital, London	John Radcliffe Hospital, Oxford	Royal Preston Hospital
Bart’s and The London NHS Trust	Belfast City Hospital	Greater Manchester Medical Centre, Manchester
Poole NHS Trust	Walton Neurological Centre, Liverpool	Ninewells Hospital, Dundee
Cambridge University Hospital	Southmead Hospital, Bristol	Queen’s Medical Centre, Nottingham
Derriford Hospital, Plymouth		Royal Victoria Infirmary, Newcastle
Southampton University Hospital		
Queen’s Hospital, Romford		

The UK MND DNA Bank was a collaborative project adopting a ‘hub and spoke’ model. Three regional Hub Centres were established at London (King’s College Hospital), Sheffield (Royal Hallamshire Hospital), and Birmingham (Queen Elizabeth Hospital) linking with a total of 16 ‘spoke centres’ spread across England, Scotland, Wales and Northern Ireland. These included hospitals that are part of the MND Association’s Care Centre Network and centres, which form part of the Department of Health/NIHR Dementias and Neurodegenerative Diseases Research Network (DeNDRoN).

The MND Association’s Biomedical Research Advisory Panel (BRAP) oversee the governance and the strategic development of the DNA Bank, ensuring that samples are utilised in an appropriate fashion, and that any clinical information requested is appropriate for the proposed study. Applications for sample use are only considered for projects which have guaranteed funding and as a result, have been peer reviewed. In addition, the Technical Access Committee (TAC) at CIGMR Biobank, determine sample requirements for the technology platform to be used, the quantity of sample required and ensure any leftover samples are returned or destroyed. All applications for access to the samples are judged on merit. Having obtained approval from BRAP and the technical access committee, applicants select samples from the DNA Bank in collaboration with the MND Association based on their requirements for specific patient cohorts e.g. gender, site of onset etc. This ensures that all the required parameters of the project are met, whilst maintaining strict governance over which samples are used. In order to receive material and clinical information from the DNA Bank, all applicants must agree to the terms and conditions of sample use (see Additional file 1). This specifies the user and specific purpose for which the samples and data are to be licensed, including standard terms as to the ownership, exploitation and dissemination of results, and requirements that the user conforms to the terms of the participants’ consent.

## Sample collection, storage and quality control

Sample collection began in 2003. All participants were over 18 years of age. In order to ensure that the patient cohort was representative of disease prognosis, patients must have experienced symptom onset (significant muscle weakness) on or after January 2002. All patients fulfilled El Escorial criteria for probable or definite Amyotrophic Lateral Sclerosis (ALS) [10]. Patients presenting with Progressive Muscular Atrophy (PMA), Primary Lateral Sclerosis (PLS) or Progressive Bulbar Palsy (PBP) [11, 12] were also included in the study. Patients were recruited by consultant neurologists with a specialist interest in MND in participating centres. Patients participating in other clinical research projects were not excluded from the study. Blood samples were also collected from consenting partners/carers, providing some degree of matching in terms of age, education, environmental exposure and often ethnicity. Where patients presented with familial MND, blood samples were collected from family members for linkage analysis. Where patients presented with sporadic MND, where possible, blood samples were also collected from parents or from a parent and sibling, to give so-called ‘Trio Samples’ increasing the amount of genetic information available for researchers.

Informed consent to participate was sought from all patients, family members and controls. Ethical approval for the collection of samples and the creation of the UK MND DNA Bank was given by the Trent Research Ethics Committee in February 2003 ref MREC/02/4/107 and in July 2009, ref 09/HO405/32. Participants were provided with detailed information and contact details and could withdraw from the study at any time. The samples were pseudo-anonymised and an online bespoke clinical database was developed to facilitate data entry and collection by the research nurses and enable tracking of trends in clinical parameters such as symptom onset and presentation for data analysis. Storage and access to this data set is in accordance with the UK Data Protection Act 1998 [13]. As custodians of the DNA Bank all enquiries for access to the clinical data or about the database itself must be directed through the MND Association.

Prior to 2010, DNA extraction from donated blood samples was carried out at individual Hub centres using the Nucleon BACC3 protocol (Amersham, UK). Extracted DNA was sent to CIGMR Biobank for long-term storage. On receipt, all DNA samples were run on 1 % agarose gels alongside molecular weight markers of appropriate size to check integrity. From August 2010, DNA extraction was carried out at CIGMR Biobank using automated robotic processing under ISO900:2000 operating standards. In all cases, both when imported from Hub centres, or extracted by CIGMR Biobank themselves, DNA concentration was measured using a nanodrop spectrophotometer. Samples with OD ratios outside the normal range were removed from the cohort and contaminants washed using ethanol precipitation. Final DNA concentration was measured using Quant-iT™ Picogreen® dsDNA Assay Kit (Invitrogenc™ Life Technologies, UK). Samples were measured on 96 well plates, in triplicate against standards of known concentration for quality control.

DNA aliquots are stored in 2D bar coded tubes for sample tracking purposes. A relational database recorded the 2D barcodes associated with each patient/donor ID. All samples within the collection were screened for gender using PCR on presumed duplicate samples according to standard protocols. Samples with a mismatch between the expected gender as recorded in the patient information, and actual gender as confirmed by PCR, were rescreened using an alternative PCR method of gender identification based on the absence/presence of *Alu* sequence [14]. Any samples with a confirmed discrepancy were ring fenced from the collection and suspended from the in-house laboratory management system.

Peripheral blood lymphocytes (PBLs) were isolated from whole blood samples at ECACC using density gradient centrifugation. An aliquot of untransformed PBLs was stored in liquid nitrogen for safekeeping, whilst the remaining PBLs were transformed using the Epstein Barr virus according to standard protocols [15]. As part of routine quality assurance, all cell lines were screened for Mycoplasma contamination, and a proportion of every batch tested for sterility, cell count and viability. Authenticity against source material (blood spot card prepared at receipt) using STR-PCR profiling confirmed that no sample mix ups had occurred during processing. The resulting lymphoblastoid cell lines were cryopreserved and are used to restock the DNA Bank when stock levels become low.

## The UK MND DNA Bank

**Fig. 1:**
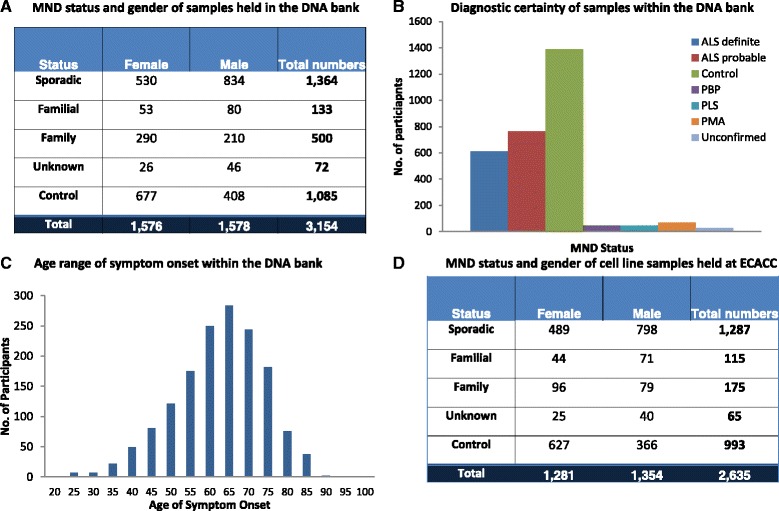
The UK MND DNA Bank. The UK MND DNA Bank comprises 3159 high quality DNA samples. 1344 samples were taken from individuals diagnosed with sporadic MND (**a** and **b**). There were 133 familial MND samples within the collection and a further 500 samples taken from family members, including samples that form 28 parent trio sets and 27 sibling trio sets. The remaining 1085 samples were taken from controls. In line with previous findings, where MND has been diagnosed, the breakdown of gender in the collection is around 60 % male (**a**). The average age of onset was approximately 62 years of age (**c**). In total 2653 frozen lymphoblastoid cell lines are held in storage at ECACC. Of these 1267 samples were generated from whole blood taken from patients with sporadic MND. 115 cell lines were generated from familial samples and the remaining 1058 cells lines have been established using blood samples obtained from control or family members (**d**)

**Fig. 2:**
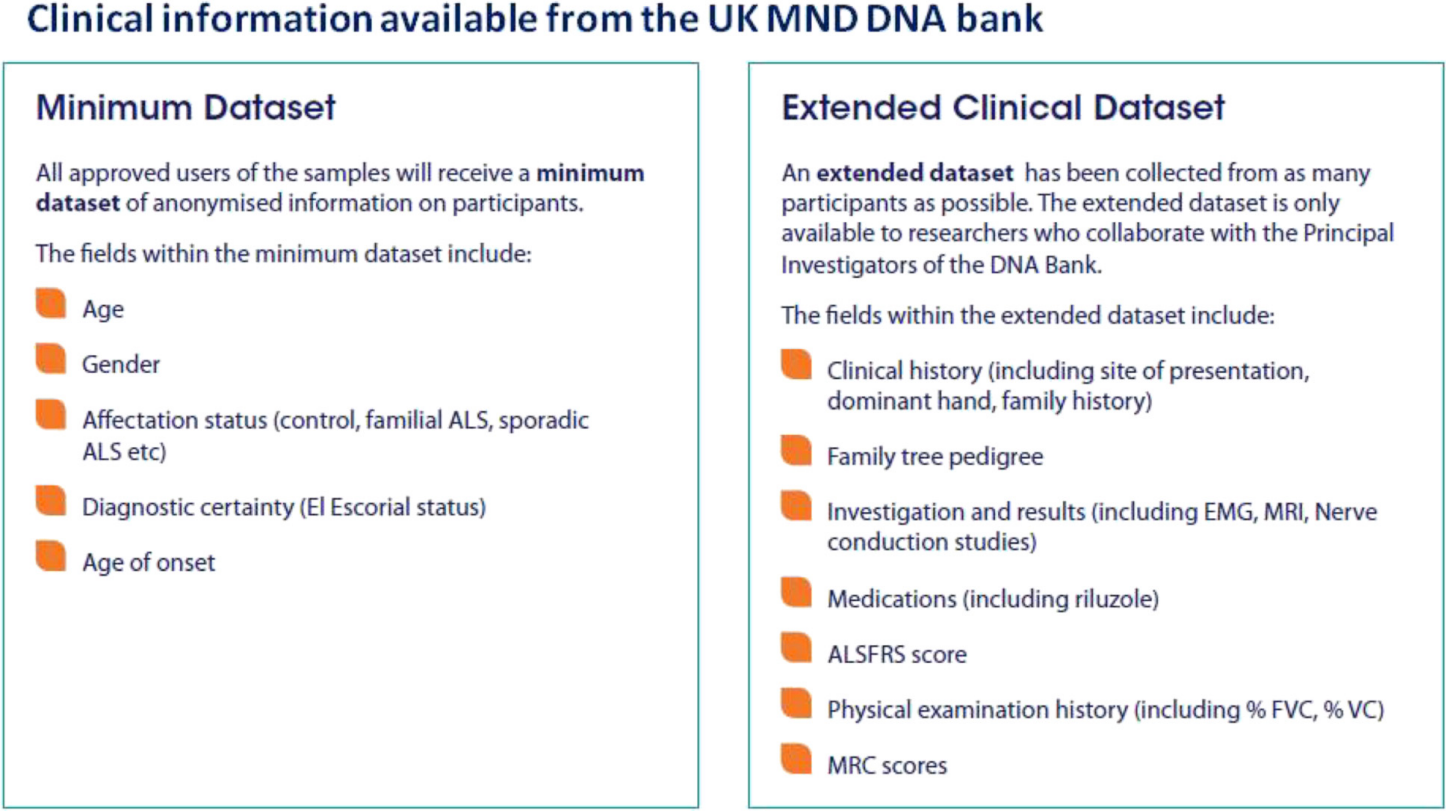
Clinical information available from the UK MND DNA Bank

In October 2012, at the end of the collection period, the UK MND DNA Bank comprised 3159 high quality DNA samples. Of these, 1344 samples were taken from individuals diagnosed with sporadic MND (see Fig. 1a and 1b). There were 133 familial MND samples within the collection and a further 500 samples taken from family members, including samples that form 28 parent trio sets and 27 sibling trio sets. The remaining 1085 samples were taken from controls. In line with population-based demographic for the disease [16] the breakdown of gender in the collection is around 60 % male (Fig. 1a). The average age of onset was approximately 62 years of age (Fig. 1c). Each sample is accompanied by a minimum dataset of: age at which the samples were taken; gender; disease status; and where appropriate diagnostic certainty (El Escorial Status) and age of onset (calculated from date of birth and date of symptom onset). An extended dataset has been collected for as many participants as possible (see Fig. 2) but it is not a complete dataset for the entire collection. Data varies greatly for each characteristic, for example, data such as site of onset and dominant hand has been taken for around 97 % of all patient participants, whilst Riluzole usage has only been noted for around 85 % of all patient participants. In total 2653 frozen lymphoblastoid cell lines are held in storage at ECACC following a PBL transformation success rate of 97 %. Of these, 1267 samples were generated from whole blood taken from patients with sporadic MND. 115 cell lines were generated from familial samples and the remaining 1058 cells lines have been established using blood samples obtained from control or family members (see Fig. 1d). Researchers are able to access transformed cell lines in collaboration with the Principal Investigators of the DNA Bank for use in genomic research projects approved by BRAP.

Each sample withdrawn from the UK MND DNA Bank is accompanied by a minimum dataset of: age at which the samples were taken; gender; disease status; and where appropriate diagnostic certainty (El Escorial Status) and age of onset (calculated from date of birth and date of symptom onset). An extended dataset has been collected for as many participants as possible but it is not a complete dataset for the entire collection. The clinical information was collected by the Research Nurse using a brief clinical questionnaire. Identifying data was kept at each Hub centre in secure locations in accordance with the Data Protection Act.

Table 2 shows the success rates for PCRs performed on the DNA samples within the collection. The failure rate of the quality control assay was less than 1.5 %, suggesting that the quality of DNA within the collection is very high. The gender results from these assays were directly compared to the gender recorded for individuals on the clinical database. Where there was a discrepancy between the expected gender and that determined in the assay, patient clinical notes were rechecked. In the absence of a clerical error, samples were rescreened using both the original AMEL marker and an alternative gender marker, the Human ALU expansion [14]. In total, 3,415 individual samples were screened to confirm gender.Sixty two samples that continued to show a discrepancy between the expected gender and the assay gender were ring fenced from the collection and suspended from the laboratory management system.

**Table 2:** Quality control PCR assay fail rate

Type of assay	No. samples screened	% assay fail
Abi Identifier Kit - AMEL Marker	768	1.30
Gender based PCR - AMEL marker	2750	0.62

Quality control assays were carried out across the collection. Sixty two samples showed a continued discrepancy between the gender of the actual DNA sample and that stated in the clinical notes and were ring fenced from the collection.

The UK MND DNA Bank was designed to be an international research resource with the fundamental guarantee that it would supply high quality DNA samples with good integrity and accompanying high quality clinical data. Establishing the resource was challenging and understandably the DNA Bank does have limitations. DNA samples from the bank represent an incident not prevalent population and are unlikely to be biased however, the genomic DNA supply itself is limited and although cell lines have been established, the DNA from such cell lines may have sequence changes compared with the original genomic samples and be unsuitable for use in some applications [17, 18]. This fact must be considered when choosing to use cell line derived DNA even if the DNA itself is of a high standard as demonstrated by the rigorous quality control assays in place. In addition, genomic DNA taken from the blood may not be entirely representative of the disease. It is possible that causative gene mutations for sporadic MND are somatic and as a result are found only in the cells of the central nervous system that are affected by disease and would therefore not be present in the blood samples provided [19]. Researchers would need to directly compare genomic DNA from the central nervous system with that from the UK MND DNA Bank to completely resolve this issue. Similarly, researchers who request access to transformed cell lines for use in genomic based research are also advised to carefully validate cell lines both at the start and end of their project using robust techniques such as STR profiling. Transformed cell lines may have genetic changes and rearrangements, and cell lines themselves can show genetic instability and phenotypic drift through prolonged culture. Cell line authentication is in accordance with the guidelines published by the International Cell Line Authentication Committee (ICLAC) [20] and is well documented as part of the terms and conditions of sample use.

Obtaining high quality clinical data also presents a problem for the DNA Bank. The extended dataset for the collection is extremely valuable but is incomplete. Clinical information is dynamic and changes over time. Whilst it is possible to access patient records and update the dataset for some fields, such as to record the date of death of a patient to provide information about survival, or to update results from gene screens, this is not possible for all fields within the database and can be a complicated process. Having a clearly defined minimum dataset from the beginning helps manage this constraint, but deciding what parameters should be included in the minimum dataset is difficult; the pertinent data of the future may not be the same as today and as a result some enquiries from researchers will always end in frustration.

As part of the governance of the DNA Bank, the MND Association must ensure compliance with legal and regulatory requirements. The Association must also guarantee that the resource adheres to rigorous research standards and is used in the further understanding of motor neuron disease; this includes prioritising access to those parts of the DNA Bank that are limited in availability, clarifying intellectual property rights and disseminating the results that flow from it.

To date more than twenty projects have withdrawn samples from the DNA Bank. DNA samples have been used in complex, technical protocols such as genotyping, gene sequencing and genome-wide association studies and numerous papers have been published or are in press [21–32]. Importantly, projects using samples from the DNA Bank have directly led to the detection of several MND causing genes including *C9orf72* and more recently *Tub4A* [21, 23, 28, 29, 32]. With researchers now encouraged to publish in an open access format as part of the DNA Bank governance, and to deposit data from sequencing projects within accessible databases such as ALSOD: the Amyotrophic Lateral Sclerosis Online Database [7] and European Genome-Phenome archive [33], the dissemination and discussion of results by the research community is ensured. In 2014 a proposal to perform whole genome sequencing on DNA samples from the UK MND DNA Bank as part of the international collaboration called Project MinE [34] was approved. This exciting project will allow Next Generation Sequencing data to be collected from DNA Bank samples and shared across research groups. The data will also confirm the accuracy of existing studies through imputation. It is hoped that sequencing DNA Bank samples will allow the identification of rare variants responsible for sporadic disease, continuously widening our knowledge about how genetic changes can contribute to MND.

DNA Bank cell lines have also been used in a variety of genomic projects investigating the effect of specific gene mutations on RNA regulation and protein expression [35, 36]. This unexpected demand for the cells has forced the DNA bank to consider the future potential of this resource, which was originally only meant for re stocking valuable DNA. Looking to the future, it is likely that master and working cell banks will be created for the most valuable cell lines in order to manage demands on the cell lines whilst also maintaining the high standards of the collection. In addition, ethical approval to extend the use of the cell lines beyond their original scope of genomic research was granted in 2014 by the Derby-East Midlands Research Ethics Committee ref no. 14/EM/1088. This change in permission will potentially allow researchers to generate primary neuronal cultures and highly desirable induced pluripotent stem cell (iPSC) lines either from the current cell lines stored at ECACC, or from the original untransformed peripheral bloody lymphocytes [37]. The iPS cell lines could act as new disease models for drug screening and other potential treatments, as well as acting as tools for analysing downstream mechanisms involved in disease pathogenesis. Clearly the role of the DNA Bank in the governance of such samples will be paramount; it is simply not enough to provide high quality samples, but following how those samples have been used and ensuring the results are disseminated and discussed is the only way to ensure research continues to move forward.

The DNA Bank is the only national UK biobank specifically created for the collection, storage and distribution of MND samples. Other biobanks have been created for rare diseases or more specifically for neurodegenerative diseases but in all cases the number of MND samples actually available from the biobank can be fairly limited. Details of European biobanks and repositories available for MND researchers are documented on the AriSLA ALScience webpage [38]. International multicentre ALS studies are beginning to bring together patients registered in neurology clinics across countries in a bid to work together. In 2006 the Japanese Consortium for Amyotrophic Lateral Sclerosis (JaCALS) started recruiting patients with ALS to a multicentre study. Genomic DNA samples and B-cell lines from patients with ALS are stored and linked to their clinical information in a model fairly similar to the one we have followed. Clinical research coordinators check patients’ scores on the ALS Functional Rating Scale-revised and their prognoses every 3 months via a telephone survey [39]. Such collaborations will ultimately help coordinate collections of MND specific samples across countries and hopefully in the future the lack of good quality MND samples may not present the problem it once did. With large international research collaborations such as Project MinE now more common place it is clear that having access to such samples will be hugely important to this field of research; for this reason alone the UK MND DNA Bank is clearly a hugely important resource. The original scope of the DNA Bank was to make a quantal difference in our understanding of MND and it is well on the way to fulfilling this promise.

## Additional file

Additional file 1:
**Terms and conditions for sample use from the UK MND DNA Bank.pdf.**

## Abbreviations

MND: Motor Neurone Disease
ALS: Amyotrophic Lateral Sclerosis
ECACC: European Collection of Cell Cultures
CIGMR: Centre for Integrated Medical Research
BRAP: Biomedical Research Advisory Panel
TAC: Technical Access Committee
PMA: Progressive Muscular Atrophy
PLS: Primary Lateral Sclerosis
PBP: Progressive Bulbar Palsy
PBL: Peripheral Blood Lymphocyte
ICLAC: International Cell Line Authentication Committee
