# Supplementary material for: Establishing the UK DNA Bank for motor neuron disease (MND)
Source: BMC Genet. 2015 Jul 14;16:84. doi: 10.1186/s12863-015-0236-6 (PMC4501191; doi:10.1186/s12863-015-0236-6)
Supplement: Additional file 1: — Terms and conditions for sample use from the UK MND DNA Bank.pdf. [file 12863_2015_236_MOESM1_ESM.docx]

| 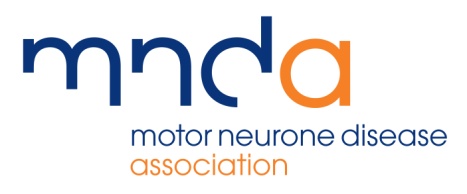 | 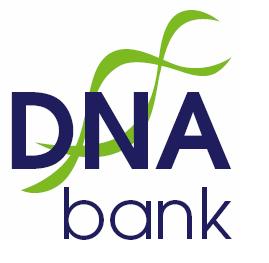 |
| --- | --- |

## Terms AND Conditions for Sample Use

**Background**

The UK MND DNA Bank (hereafter referred to as ‘the DNA Bank’) has been established to provide the international research community with a resource to identify and understand causative and disease modifying factors in motor neurone diseases. It was funded through grants provided by the MND Association and the Wellcome Trust.

Requests for information on the samples available should be made to Lucy Smith, Research Information Administrator at the MND Association ([dnabank@mndassociation.org](mailto:dnabank@mndassociation.org) ).

The MND Association acts as custodian of the material and clinical information. The principles of the custodianship are:

- Protect participants, honour commitments made to them and act within the scope of their consents
- Ensure compliance with legal and regulatory requirements
- Ensure that the resource adheres to highest research standards and is used to further understanding of motor neurone disease
- Prioritise access to those parts of the DNA Bank that are limited in availability
- Clarify intellectual property rights and the results that flow from the DNA Bank.

Subject to these constraints, the MND Association will encourage and provide access to the DNA Bank and the results that flow from it as widely and openly as possible in order to maximise its use and value for research. This will include access from the academic, commercial and public sectors in the UK and elsewhere.

The MND Association has established partnerships with CIGMR Biobank (formerly known as Biobanking solutions (BBS)) and the European Collection of Cell Cultures (ECACC).

Based at the University of Manchester, and forming part of the Centre for Integrated Genomic Medical Research (CIGMR), CIGMR Biobank manages access to DNA samples in the permanent collection of the DNA Bank. Where there are low yields of genomic DNA in the collection, users may be offered cell line DNA as an alternative.

As one of the Public Health England collections, ECACC manage and store EBV-transformed lymphocytes and peripheral blood lymphocytes, derived from blood samples from participants.

1. **General considerations**
   1. In order to receive material and clinical information from the DNA Bank, all applicants must agree to abide by the Terms and Conditions listed in this document.
   2. The MND Association and its partners will only authorise the release of samples and data to researchers where a compelling case can be made to justify their use.

*Access to cell lines*

- 1. All access to EBV-transformed cell lines and peripheral blood lymphocytes are restricted to studies specifically investigating the aetiopathogenesis of, and the development of effective treatments for, motor neurone disease and related neurodegenerative disorders, including frontotemporal dementia.
  2. Where access to create induced pluripotent stem cells (iPSCs) is requested, access is limited to the use of iPSCs to create cells affected in motor neurone disease, for example the creation of motor neurones and glial cells. Access will not be permitted for the creation of cells unaffected in motor neurone disease.
  3. Access to cell lines is limited to researchers based within the UK.

1. **Applications**
   1. **All enquiries and requests must be made via the MND Association**.
   2. Requests will be considered for access to DNA, cell pellets, EBV-transformed cell lines and peripheral blood lymphocytes.
   3. Application for access to material from the DNA Bank will be treated as confidential and will be considered by:

- the MND Association’s Biomedical Research Advisory Panel (BRAP), where the Principal Investigators (PIs) of the DNA Bank (Professors Ammar Al-Chalabi, Karen Morrison and Pamela Shaw) are co-opted onto the Panel for these discussions
- The CIGMR Biobank Technical Access Committee (TAC). The TAC will only be consulted for requests for DNA stored at CIGMR Biobank
  1. The cell lines stored at ECACC represent a subset of the samples where genomic DNA has been collected. Specifically, cell lines are not available for family members of people with sporadic MND.

*BRAP*

- 1. The BRAP (present Chairman Professor Mike Stewart, The Open University) is appointed by the MND Association’s Board of Trustees. The panel’s overall function is to oversee the MND Association’s biomedical research activities and provide strategic guidance, ensuring good governance.
  2. With regard to the DNA Bank, the BRAP’s function is to oversee the governance and the strategic development of the DNA Bank, ensuring that samples are utilised in an appropriate fashion, and that clinical information requested is appropriate for the proposed study.

*TAC*

- 1. The TAC will verify that the type and quantity of DNA required is reasonable, feasible and appropriate for the type of study and the technology platform to be used. It will also seek to ensure that any leftover samples are returned or destroyed.

*Access process*

- 1. The BRAP will consider requests for access to i) samples: including DNA, EBV lymphoblastoid cell lines or peripheral blood lymphocytes (a standard, minimum dataset of phenotype will be provided with all samples); ii) additional phenotypic data and iii) genotype data.
  2. All initial approaches for sample use will be considered by the BRAP. They will judge the scientific merit of the proposed work and its relevance to MND, novelty/innovation and track record of the applicants.
  3. If DNA from CIGMR Biobank has been requested, on the BRAP’s favourable opinion, the application will be forwarded to the TAC. On their favourable assessment the release of the samples from CIGMR Biobank will be authorised.
  4. The BRAP will review requests for access at least every three months.
  5. Generally, the MND Association will permit the user up to a specified, time limited period, for use of samples in approved studies, from the date of release of samples/data.
  6. An extension of use may be permitted, upon request, where circumstances merit it. Such a request must be made to the MND Association either at the point of application or within two calendar months before or after the original expiry date.

*Prioritisation of requests*

- 1. The DNA Bank will be managed in order to optimise its use and value for research.
  2. The MND Association will not offer access to any particular part of the DNA Bank to any single user or group of users on an exclusive basis, though access to the depletable aspects of the DNA Bank may necessarily be limited. All applications for access to the DNA Bank will be judged on merit.
  3. Prioritisation and approval of requests for access to samples and clinical data will be determined according to criteria to be set by the MND Association on advice from the BRAP.

1. **Conditions of access**

*Funding*

- 1. Applicants must have the necessary funding for the research they are intending to undertake.
  2. Provisional access may be obtained prior to securing funding. However samples will not be released prior to obtaining funding.
  3. If funding is obtained on the basis of claiming access to samples, before provisional access has been granted, applications for access may be rejected.
  4. Evidence of funding, for example a grant offer letter, should be provided before the release of samples.
  5. The applicant must inform the MND Association as soon as possible of any significant changes to the logistics of the study (such as change of key personnel or location of research).
  6. Where an application for ***funding*** to use the samples in the DNA Bank is submitted to the MND Association, the normal process for funding applications will be followed (see our Research Governance Overview document on our website for further information). A request for access to DNA Bank samples must be submitted separately in these cases.

*Peer review*

- 1. All scientific proposals in which samples or data from the DNA Bank will be used should be peer reviewed. Evidence of peer review will usually be in the form of receipt of independent funding for the proposal.
  2. In the absence of this evidence the MND Association may seek further information from the applicant or subject the proposal to our own peer review. The confidentiality of the proposal will be respected at all times.

*Fees*

- 1. Applicants must meet all costs connected with supplying (and destroying) material from the DNA Bank.
  2. Researchers accessing samples from CIGMR Biobank or ECACC will pay the standard costs for preparation and shipping of samples. Quotations can be provided on request to Debbie Payne at CIGMR Biobank, and Debbie Blick at ECACC respectively.

Their contact details are:

Debbie Payne, CIGMR Biobank, University of Manchester; Debbie.Payne@manchester.ac.uk; 0161 275 1625

Debbie Blick, Blood Processing and Transformations Supervisor, Human Genetic Services, Culture Collections, Public Health England Porton Down; Debbie.Blick@phe.gov.uk; 01980 612611

- 1. Costs for access to academic and public researchers will be kept at a minimum, to encourage use.
  2. The MND Association may charge an administration fee and a surcharge per sample to commercial users. However, consideration will be given to the impact of charges on the full range of potential users and uses including, for example, collaboration between academia and industry, smaller companies or innovative use in larger companies.

*Compliance with regulations*

- 1. Applicants must provide documentary evidence that their proposal has the approval of and complies with the relevant ethical or regulatory bodies (non-UK institutions must refer to their national regulations, as required).

Specifically:

- 1. All applicants must have ethical approval for the research they are planning to perform.
  2. All applicants must ensure that human tissue licences are in place when using ‘relevant material’^[[1]](#footnote-1)^ as defined by the Human Tissue Authority.

*Access to data accompanying samples*

- 1. Under no circumstances should the applicant, or anyone acting on their behalf, attempt to identify or contact any of the participants.
  2. Information identifying participants will not be available to users for research. All applicants will receive the minimum dataset on any samples to which they are given access (See appendix for details of the minimum dataset).

The process for requesting data from the extended dataset (of phenotypic information) or genetic classification data is given below.

*Access to data within the extended dataset*

- 1. Research studies requiring additional phenotypic or genetic classification data may only be conducted in collaboration with the PIs of the DNA Bank (see 2.3). Requests to access such data should be made in the application for access to the samples.
  2. Access to the extended dataset will only be permitted for research that meets the Terms and Conditions for access laid out in this document.
  3. These data were acquired from participants where possible, it is not a complete dataset for all participants. Thus requesting data from the extended dataset may restrict the number of samples available.
  4. Family pedigree data linking family member samples to the index sample of a participant with MND form part of the extended dataset, thus samples from family members are only available to researchers collaborating with the PIs of the DNA Bank.

1. **General Conditions**
   1. Applicants must not perform any research on material from the DNA Bank that was not agreed as part of their initial application, without permission, in writing, from the MND Association.
   2. Any changes to the original approval for the use of samples must be approved and documented by a DNA bank amendment form.
   3. Applicants must not release any material from the DNA Bank to third parties.
   4. Applicants must return or destroy any material not used in their research after the designated period, unless specific consent for retention has been obtained, in writing from the MND Association.
   5. Applicants must accept full responsibility for all aspects of safety concerning the storage and use of material they receive. The MND Association and their respective agents do not accept any responsibility for loss or injury caused by use of any material supplied.
   6. Applicants receiving samples from CIGMR Biobank must adhere to their Terms and Conditions^[[2]](#footnote-2)^.

*Cell Lines*

- 1. Applicants receiving samples from ECACC must adhere to their Terms and Conditions^[[3]](#footnote-3)^.
  2. Applicants are strongly advised to confirm the authenticity of cell lines at the start and at end of the project prior to publication, to ensure that no mix up or contamination of cell lines has occurred throughout the project lifetime. This is in accordance to guidelines set out by the International Cell Line Authentication Committee (ICLAC)^4^.
  3. Failure to comply with the Terms and Conditions may result in the application of sanctions to the applicant, not limited to but including: restrictions on future access and notification to pertinent funding bodies, the researcher’s Institution and journal editors.

1. **Dissemination**

*Details of access approved*

- 1. Details of approvals for access will be treated in the utmost confidence until publication of results by successful applicants. However, the MND Association reserves the right to publish the title of the project for which access to the resource has been granted.
  2. The timing of this publication will be after samples have been released and prior to the identification and/or publication of any results. Users will be consulted in advance and may suggest modification of titles to preserve future confidentiality, intellectual property (IP) rights or protect commercially sensitive information.

*Progress reports*

- 1. **Users are required to provide the MND Association with at least annual updates on their use of samples approved from the DNA bank.** These updates are checked and signed off by the Head of Research at the Association (if funding to conduct the research has been sourced externally) or as part of their annual funding progress reports by two members of the BRAP (if funding is provided by the Association).
  2. Annual updates on the use of the samples are provided to the BRAP and to the Association’s Board of Trustees.
  3. **Users will notify the MND Association of their progress within six months of expiry date of sample usage, by completion of a proforma report form.** It is recognised that a delay prior to dissemination is often necessary in order to enable a larger project to be completed, a patent to be filed or other competitive advantage to be pursued.
  4. The MND Association must be informed as to the reason why full details cannot be communicated in the report form and users will agree to notify the MND Association of their results by completion of a further report form at the earliest opportunity.

*Dissemination of results*

- 1. Users will disseminate the results of their research as rapidly and widely as possible. They will be encouraged to discuss their research with other scientists and the public and to share relevant data and materials as openly as possible.
  2. Authors of peer reviewed papers, directly reporting the results from the use of the DNA bank, are eligible to apply for the payment of open access fees by the MND Association. Such research papers should be available within the Europe PubMed Central (Europe PMC) repository as soon as possible, but definitely within six months of publication of the paper (for more information see [www.mndassociation.org/openaccess](http://www.mndassociation.org/openaccess)).
  3. The MND Association should be notified of any dissemination of data or results resulting from the supply of samples (see also Section 7 Data Sharing).
  4. In order to assess the utility and impact of the DNA Bank and facilitate further research, the MND Association will hold details of all published findings and published patent applications.
  5. Reprints of publications arising from use of the resource must be sent to the MND Association when available. If necessary, users should also provide additional details of their techniques so that other researchers will be able to comprehend the results.

1. **Acknowledgements**
   1. Applicants must acknowledge supply of samples from the DNA Bank in all methods of dissemination of the research arising from use of material.
   2. The acknowledgement should include the participants who donated samples to the DNA Bank, clinicians of the participating collection centres and the funders for the collection as outlined below.
   3. All participating clinicians are acknowledged on the MND Association’s website, citation of this link may be used as a method of acknowledging their contribution.
   4. Authorship on papers is only appropriate where contributors fulfil at least three criteria set out by the International Committee of Medical Journal Editors: (i) substantial contributions to conception/design; (ii) acquisition of data; (iii) analysis/interpretation of data; (iv) drafting or critical revision of the article; (v) final approval of the version to be published. Minimum standards for three criteria must be fulfilled.
   5. The MND Association will provide additional guidance if required.
   6. All publications should include the following wording in the Acknowledgements Section: ‘**Samples used in this research were entirely/in part obtained from the UK MND DNA Bank for MND Research, funded by the MND Association and the Wellcome Trust. We would like to thank people with MND and their families for their participation in this project**’.
   7. If appropriate the services of CIGMR Biobank should also be acknowledged, using the following wording: **‘We acknowledge sample management undertaken by CIGMR Biobank at the Centre for Integrated Genomic Medical Research, University of Manchester’**
2. **Data Sharing**
   1. All data generated as a result of access to samples within the DNA Bank should be made available to the wider research community by depositing the data within the European Genome-Phenome Archive (EGA) [www.ebi.ac.uk/ega](http://www.ebi.ac.uk/ega) .
   2. Data should be deposited on first publication or release of the data, whichever is the soonest.
   3. Access to the datasets deposited within the EGA is governed by a MND Collection Data Access Committee.
   4. The MND Collection Data Access Committee consists of the BRAP of the MND Association, the PIs of the DNA Bank and one representative of the generators of each dataset deposited.
   5. On deposition of data at EGA, researchers should nominate one representative for membership of the Data Access Committee to the MND Association.
   6. Data generator representatives of the Data Access Committee will ***only*** be consulted when applications to use data generated from their research are considered.
   7. The need for data generators to be appropriately credited for their scientific contribution and investment in data generation will be recognised. It is therefore expected that all data users both honour agreements in line with the Fort Lauderdale Principles on data sharing^[[4]](#footnote-4)^ and appropriately acknowledge the contributions and intellectual property (IP) of others.
   8. It is the intention of the PIs to conduct whole genome sequencing (WGS) on the DNA Bank samples. Therefore, applications for WGS will only be considered if they are in collaboration with the PIs of the DNA Bank.
3. **Intellectual Property relating to research using the resource**
   1. Intellectual Property (IP) arising out of research using the resource will vest in the investigator creating it, their Institution, or in appropriate cases, their assignees.
   2. The MND Association will require users to have in place suitable arrangements to ensure that IP arising from their use of the resource vests in them, their employing institution, or their assignees, and is properly identified, managed and exploited.
   3. Where the research involves a number of users and/or Institutions, suitable arrangements to manage any IP arising from the collaboration should be established in advance. This may not necessarily involve negotiating detailed agreements, but there should, as a minimum, be agreement on key terms where possible. Generally, the detailed arrangements will involve the identification of a lead institution for IP management purposes or, if appropriate, the transfer of the management or control of the IP to a third party. Before this is effected, the third party will agree to be bound by the same requirements.
   4. The IP holder is expected to vigorously pursue the development and exploitation of IP that flows directly or indirectly from the research. The MND Association reserves the right, but not the duty, to step in to exploit IP in suitable cases if, after a period of time, the lead institution chooses not to do so. Six months notice of the intention to ‘step in’ will be given before proceeding.
4. **Notification of patent applications**
   1. We require users to provide the MND Association with details at the point of filing, publication and granting of patent applications that are based on the results of any research carried out using samples or data from the DNA Bank. Information regarding filing of patents will be kept confidential. This includes arrangements for IP in studies that involve the use of additional information, biological samples or otherwise go beyond the scope of the DNA Bank.

**Appendix 1: Minimum Dataset**

| 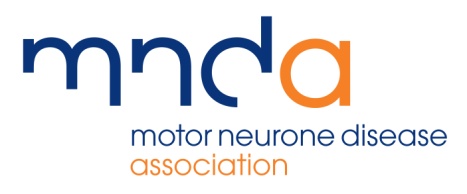 | 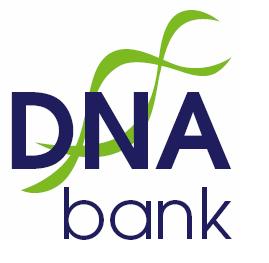 |
| --- | --- |

**Minimum dataset of UK MND DNA Bank**

All approved users of the samples from the DNA Bank will receive a minimum dataset of anonymised information on participants.

The minimum dataset includes the following information, as finalised and agreed by the DNA Bank Management Committee at their meeting in September 2006.

- age
- gender
- affectation status (control, familial ALS, sporadic ALS etc)
- diagnostic certainty (El Escorial status)
- age of onset

1. A list of relevant material according to the Human Tissue Authority is given on their website: <http://www.hta.gov.uk/legislationpoliciesandcodesofpractice/definitionofrelevantmaterial/listofmaterialsconsideredtoberelevantmaterialunderthehumantissueact2004.cfm> [↑](#footnote-ref-1)
2. Included within the CIGMR Biobank application form. [↑](#footnote-ref-2)
3. <http://www.phe-culturecollections.org.uk/orderinginfo/terms.jsp>

   ^4^ <http://iclac.org/resources/advice-scientists/> [↑](#footnote-ref-3)
4. Sharing Data from Large-Scale Biological Research Projects: A System of Tripartite Responsibility, Report of a meeting organised by the Wellcome Trust and held on 14-15 January 2003 at Fort Lauderdale, USA (aka Fort Lauderdale Principles): www.genome.gov/pages/research/wellcomereport0303.pdf [↑](#footnote-ref-4)
